# Alleviation of Lipopolysaccharide-Induced Acute Respiratory Distress Syndrome in Rats by Yiqi Huayu Jiedu Decoction: A Tandem Mass Tag-Based Proteomics Study

**DOI:** 10.3389/fphar.2020.01215

**Published:** 2020-08-28

**Authors:** Chang-yong Luo, Yan Li, Xin Li, Xu Liang, Qian Wang, Yuan-hong Ma, Cai-hua Xiong, Yan-peng Zeng, Wei Sun, Xin Wang

**Affiliations:** ^1^ College of Traditional Chinese Medicine, Beijing University of Chinese Medicine, Beijing, China; ^2^ Education Section, Dongzhimen Hospital of Beijing University of Chinese Medicine, Beijing, China; ^3^ Institute of Basic Medical Sciences, Academy of Medical Science, Peking Union Medical College, Beijing, China; ^4^ Biological Spectrum Institute, Guangdong Junfeng BFS Technology CO, Guangzhou, China

**Keywords:** Yiqi Huayu Jiedu Decoction, acute respiratory distress syndrome rat, lipopolysaccharide, proteomics, immune system, phagocytosis

## Abstract

**Background:**

To study the effect of Yiqi Huayu Jiedu Decoction (YQHYJD) on protein expression in the lung tissue of acute respiratory distress syndrome (ARDS) rats and to explore the underlying molecular therapeutic mechanism of YQHYJD.

**Methods:**

Sprague Dawley rats were administered with YQHYJD by oral gavage for 1 week. The rats were injected with lipopolysaccharide (LPS) to induce ARDS. The lung injury was assessed pathologically. Differentially expressed proteins (DEPs) were screened by quantitative proteomics and analyzed using bioinformatic tools, such as Metascape and Kyoto Encyclopedia of Genes and Genomes (KEGG) mapper. DEPs were verified by parallel reaction monitoring (PRM).

**Results:**

YQHYJD alleviated the LPS-induced pathological damage of lung tissue in rats. There were 134 DEPs among the YQHYJD treatment and model groups. The Genomes pathway analyses revealed that the DEPs were closely related to immune system pathway. The mass spectrometry analysis revealed that YQHYJD exhibits a protective effect on lung tissue by significantly upregulating hematopoietic cell kinase (Hck), phospholipid phosphatase 3 (Plpp3), myristoylated-alanine rich C-kinase substrate (Marcks), and Actin-related protein 2/3 complex subunit 2 (Arpc2), which are related to Fc gamma receptor-mediated phagocytosis pathway.

**Conclusion:**

YQHYJD can alleviate the lung injury of ARDS rats by regulating the Fc gamma receptor-mediated phagocytosis pathway, which is related to immune system.

## Introduction

Acute respiratory distress syndrome (ARDS) is a clinical condition characterized by progressive hypoxemia and respiratory distress. The most common underlying condition is severe infection (e.g., sepsis/septic shock with or without severe pneumonia) due to various microbial pathogens, which include bacteria, viruses, fungi, rickettsia, and parasites. The histological characteristics of ARDS include acute diffuse inflammatory lung injury, which results in decreased barrier function of alveolar capillary endothelial cells, increased permeability, and exudation of a large amount of protein, red blood cells, and liquids. Subsequently, this leads to osmotic pulmonary edema and transparent membrane formation, which markedly affect ventilation (Thompson et al., 2017). Globally, there are more than 3 million reported cases of ARDS each year with a mortality rate of more than 40% (Fan et al., 2018). At present, the global spread of covid-19 virus, ARDS is one of the most important causes of death in covid-19 patients. Including 38 studies involving 3,062 patients with covid-19, the incidence of respiratory failure or ARDS was 19.5%, and the mortality was 5.5% (Zhu et al., 2020). Although the pathogenesis of ARDS is still unclear, the key pathological features of ARDS are enhanced number and activation of inflammatory cells in the lungs, which trigger aberrant proinflammatory cytokine release and inflammation cascades. Consequently, this causes extensive damage to the microvascular endothelial cells (Yang et al., 2018). The treatment for ARDS is mainly based on protective mechanical ventilation, restrictive fluid management, and other supportive treatments, as well as on the control of the primary disease. There is an urgent need to develop a safe and effective therapy for ARDS based on the detailed pathogenetic mechanism. One meta-analysis confirmed (Wan et al., 2018) that the combination treatment with traditional Chinese medicine (TCM) and Western medicine decreases mortality among patients with ARDS. TCM comprises various components that act synergistically to achieve multiple effects (Weng et al., 2018). Compared to the single blocker, TCM may be more effective in clinical application for the prevention and treatment of ARDS.

Yiqi Huayu Jiedu Decoction (YQHYJD) is an empirical prescription that was developed by Professor Huaitang Du, a senior traditional Chinese medicine practitioners, through years of clinical practice. YQHYJD is a traditional Chinese decoction used for treating patients exhibiting acute exacerbation of chronic obstructive pulmonary disease and mild ARDS. YQHYJD comprises 8 Chinese herbal components. Several studies have indicated that the ingredients of YQHYJD, especially *Hedysarum multijugum* Maxim, *Panax notoginseng*, and *Scutellariae radix*, exhibit anti-inflammatory activities in the lungs. One study reported that astragaloside IV in *Hedysarum multijugum* Maxim effectively inhibits lipopolysaccharide (LPS)-induced acute inflammatory responses by modulating the nuclear factor (NF)-κB and AP-1 signaling pathways (Zhang and Frei, 2015). Pretreatment with *Astragalus* polysaccharides markedly attenuated tumor necrosis factor (TNF)-α-induced upregulation of intercellular adhesion molecule 1 (ICAM-1) and vascular adhesion molecule 1 (VCAM-1), as well as augmented NF-κB translocation. Moreover, *Astragalus* polysaccharides significantly reduced apoptosis, reactive oxygen species generation, and adhesion function damage in the TNF-α-treated human umbilical vein endothelial cells. This indicated that *Astragalus* polysaccharides may be used to treat and prevent endothelial cell injury-related diseases (Zhu et al., 2013). In addition to exerting direct inhibitory effects on the proinflammatory molecules, Ginsenoside Rb1 in *Panax notoginseng* is reported to attenuate LPS-induced lung injury by inhibiting the inflammatory signaling pathway (Yuan et al., 2014). Moreover, baicalein in *Scutellariae radix* inhibits matrix metalloproteinase expression, which is partly mediated by suppressing the activation of the p38 and extracellular regulated protein kinases (ERK) signaling pathways (Chen et al., 2015). Additionally, baicalein attenuates lectin-like oxidized low-density lipoprotein receptor-1 (LOX-1)-mediated endothelial oxidative dysfunction by modulating the AMPK/PKC/NADPH oxidase/NF-κB signaling pathway. Therefore, baicalein decreases the NADPH oxidase-induced reactive oxygen species formation and dysfunction of superoxide dismutase 1 (SOD-1) (Tsai et al., 2016).

**Figure 1:**
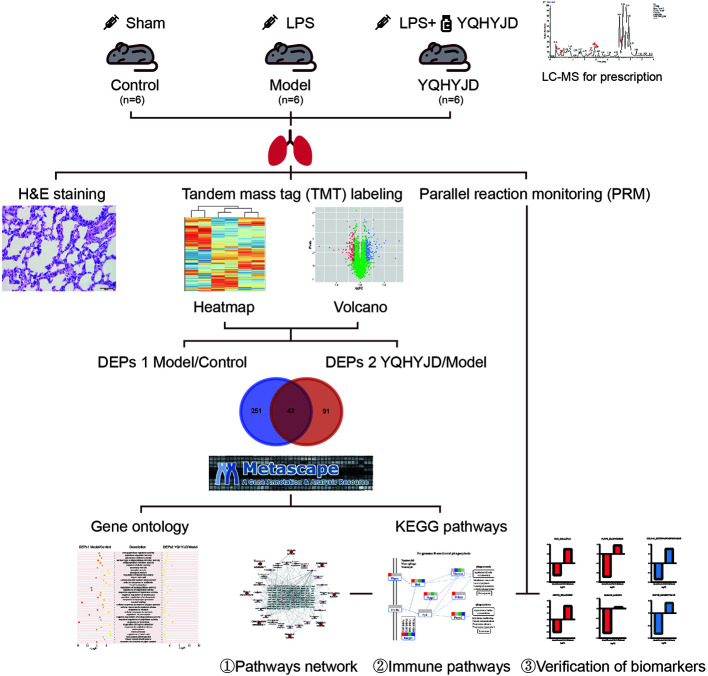
Workflow for Yiqi Huayu Jiedu Decoction (YQHYJD) intervention in Lipopolysaccharide (LPS)-Induced Acute Respiratory Distress Syndrome (ARDS). 18 Sprague Dawley rats were randomly divided into Control group, Model group, and YQHYJD group. They were intervened by Sham, LPS, LPS+YQHYJD, respectively. The quality of YQHYJD aqueous solution was controlled by Liquid chromatography-mass spectrometry (LC-MS). Hematoxylin-eosin (HE) staining was detected in lung tissue of rats in each group to determine the intervention effect of YQHYJD on LPS induced ARDS. Tandem mass tag (TMT) was performed in lung tissue of rats in each group to detect the differential proteins among groups. The target pathway and potential targets of YQHYJD were obtained by bioinformatics analysis. Finally, Parallel reaction monitoring (PRM) was used to verify the potential targets of YQHYJD on LPS induced ARDS.

Previously, we had demonstrated that YQHYJD can significantly alleviate the lung and tissue damage by decreasing the inflammatory cell infiltration and the expression of inflammatory factors, such as interleukin (IL)-1β, IL-6, IL-8, and TNF-α in the serum (Li et al., 2016; Huang et al., 2017). Furthermore, YQHYJD improved the lung coefficient and lung permeability index in the ARDS rat model and decreased the expression of key proteins involved in the proinflammatory signaling pathways, such as NF-κB and p38 (Li et al., 2017). Another study reported that YQHYJD has a protective effect on LPS-induced ARDS in rats, which may be related to enhanced expression of IL-4 and IL-10 in the serum (Luo C. Y. et al., 2019). Here, a proteomic method that conforms to the characteristics and laws of TCM was applied to further elucidate the molecular therapeutic mechanism of YQHYJD in ARDS. This study applied high-throughput proteomics technology and used rat lung tissue to analyze the differentially expressed proteins (DEPs) among the experimental groups. Additionally, the protein-protein interaction network and the genes and pathways affected by YQHYJD treatment were analyzed in the ARDS model. The key proteins were validated and the therapeutic mechanism of YQHYJD in ARDS was elucidated (**Figure 1**).

## Materials and Methods

### Reagents and Drugs

**Figure 2:**
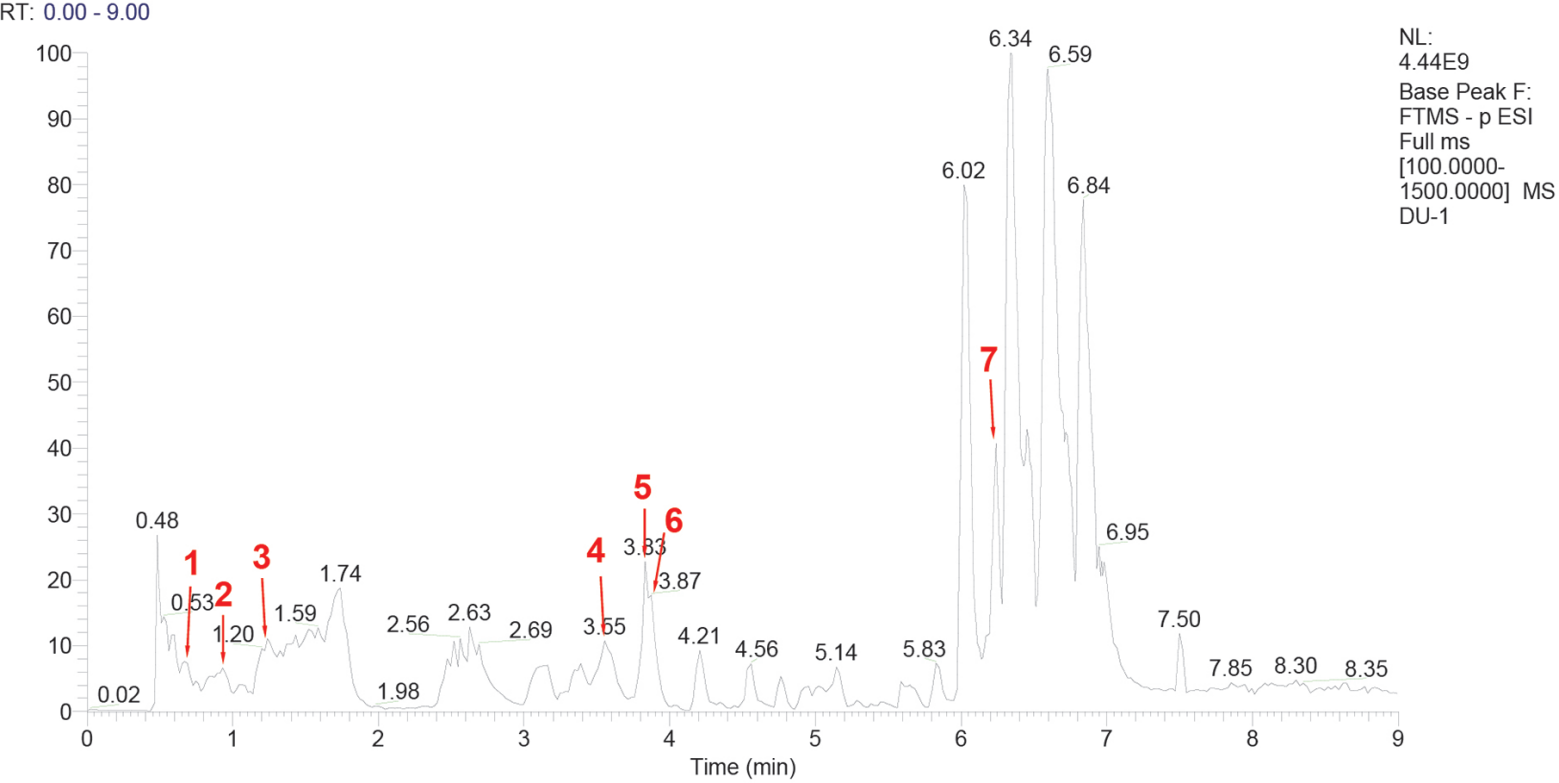
High-performance liquid chromatography-mass spectrometry (HPLC-MS) chromatogram of Yiqi Huayu Jiedu Decoction (YQHYJD) aqueous solution. The peaks 1–7 correspond to Cynaroside (1), Calycosin-7-glucoside (2), ginsenoside-Rg1 (3), ginsenoside-Re (4), astragaloside IV (5), Chlorogenic acid (6), and notoginsenoside r1 (7).

*Escherichia coli* LPS (batch number 110M4086V, 100 mg), Bicinchoninic acid kit, and tandem mass tag (TMT) labeling kit were purchased from Sigma, USA. Liquid chromatography-mass spectrometry (LC-MS) grade acetonitrile and formic acid were purchased from Fisher Chemical Company. YQHYJD comprises 8 Chinese herbal components (**Table 1**), the information of plants was collected from Kew Royal Botanic Garden (https://mpns.science.kew.org/). The herbal components were provided by the Institute of Chinese Medicine of China Academy of Chinese Medical Sciences, which were used to prepare the Chinese herbal extract (batch number: 20170320). The quality control for all the materials was validated according to the Chinese Pharmacopoeia. The LC-MS analysis was performed to characterize the YQHYJD extract (**Figure 2**). The chemical constituents of YQHYJD extract were profiled using the ultra-high-performance liquid chromatography system coupled with a high-resolution electrospray ionization mass detector.

**Table 1:** The composition and proportion of herbal components in Yiqi Huayu Jiedu Decoction (YQHYJD).

Latin name	Used Parts	Chinese Pinyin name	Weight(g)
*Astragalus mongholicus*	Roots	Huang-qi	30
*Scutellaria baicalensis*	Roots	Huang-qin	10
*Panax notoginseng*	Rhizomes	San-qi	10
*Lonicera japonica*	Flowers	Jin-yin-hua	15
*Paeonia lactiflora*	Roots	Chi-shao	15
*Mori radices*	Cortex	Sang-bai-pi	15
*Citrus × aurantium*	Fruits	Zhi-shi	12
*Lepidium apetalum*	Seeds	Ting-li-zi	15

### Experimental Animals

In total, 18 clean-grade 6-week-old male Sprague Dawley rats with an average weight of 200 ± 10 g were purchased from Beijing Vital River Laboratory Animal Technology Co., Ltd. The rats were maintained (six rats per cage) at the Barrier Laboratory of Experimental Animal Center of Dongzhimen Hospital of Beijing University of Chinese Medicine. All rats were fed with the same diet. The experiments were performed after 1-week of adaptive feeding. The animal experiments were approved by the Experimental Animal Welfare and Ethics Committee of Dongzhimen Hospital of Beijing University of Chinese Medicine (batch number: 17-08).

### Dosing and ARDS Modeling

The rats in each group were fed on a normal diet. The rats in each group were fed with normal diet. The YQHYJD group was given in the morning, once a day, at the dose of 12.2 mg/kg body weight, to prepare 1/100 body weight solution. The normal control group (Control) and model control group (Model) were given 1/100 body weight distilled water. All rats were given gavage for 7 days. The ARDS animal model was administered with LPS by a single intravenous injection to induce a systemic inflammatory response, which is the most common cause of ARDS. The phenotype of this animal model is consistent with the pathological features of ARDS lung tissue (Jiang et al., 2016). On day 7 after 6 h of gavage solution, the Control group was injected with 2 mg/kg normal saline, while the Model group and YQHYJD groups were injected with 2 mg/kg LPS solution through the tail vein. After 16 h, the rats in all three groups were allowed to fast for 8 h and dissected under anesthesia. The animals were euthanized by intra-peritoneal injection of 50 mg/kg sodium pentobarbital. The right lower lung was subjected to hematoxylin and eosin staining. The left lung was used for proteomic screening, while the right lung accessory leaf was used for mass spectrometry analysis.

### Peptide Sample Preparation and TMT Labeling

The lung tissue of each group of rats stored at -80°C was lysed and centrifuged several times. The tissue lysates of each group were stored separately. Each sample group was dispensed into 3 EP tubes. One tube containing about 30 µl sample was treated with sodium dodecyl sulfate to clarify the protein extract. In the second tube containing about 30 µl sample, protein concentration was determined by the Bradford method. The remaining sample in the third tube was subjected to protease digestion to obtain the membrane peptide. The peptide was purified by C18 column extraction. The dried peptide was quantified by the bicinchoninic acid method. The peptide was dissolved and incubated with the labeling reagent under room temperature for 2 h in the dark. The sample was vacuum dried and subjected to one-dimensional LC-MS analysis.

### Parallel Reaction Monitoring (PRM) Analysis

Eighteen lung tissue samples from three experimental groups were separately subjected to trypsin digestion. The polypeptide concentration in the digested sample was determined by the bicinchoninic acid method. Equal amount of test protein samples (10.5 µg; 0.5 µg) and iRT standard peptide was mixed to obtain a mixed sample. PRM polypeptide screening and analysis were performed using the Skyline 3.6 software. Initially, the mixed peptide samples were screened. The substrates that had a better profile in the rat lung tissue proteome database and that can be identified in the mixed sample or that have a high signal to noise ratio peak were selected for subsequent validation. Next, the selected polypeptides were subjected to PRM analysis. Eighteen samples were validated separately. The target polypeptide was verified in each sample by scheduled PRM. To ensure data quality, the mixed samples were analyzed before and after the loading of all samples and between every five samples. The instrument signal was stable throughout the analysis. Furthermore, iRT standard peptide was added to each sample to observe the stability of chromatographic retention time during the analysis. Two technical repeat analyses were performed for each sample. To reduce systematic errors, different sets of samples were randomly sequenced for mass spectrometry. In the Skyline software, the correct peaks were manually selected and all peptide results from all samples were exported and quantified.

### Statistical Analysis

The data were retrieved using the Proteome Discoverer software suite (Version 2.1, Thermo Fisher Scientific). The total proteins in the samples were obtained after data retrieval. The relative quantity of each protein in different groups was determined. The differentially expressed proteins between Model and Control are called DEPs1 and which between Model group and YQHYJD group are called DEPs2. The proteins with p-value <0.05 and absolute value of log(fold-change)(FC)>0.1 were considered as DEPs.

Metascape (https://metascape.org/gp/index.html) is used for gene ontology (GO) enrichment analysis and Kyoto Encyclopedia of Genes and Genomes (KEGG) pathways enrichment analysis of DEPs. The R Version 3.5.1 was used for graphical display. P-value scoring and Gene proportion are used to select important GO enrichment analysis and KEGG pathways.

## Results

### YQHYJD Treatment Attenuated Pathological Damage of Lung Tissue in ARDS Rats

**Figure 3:**
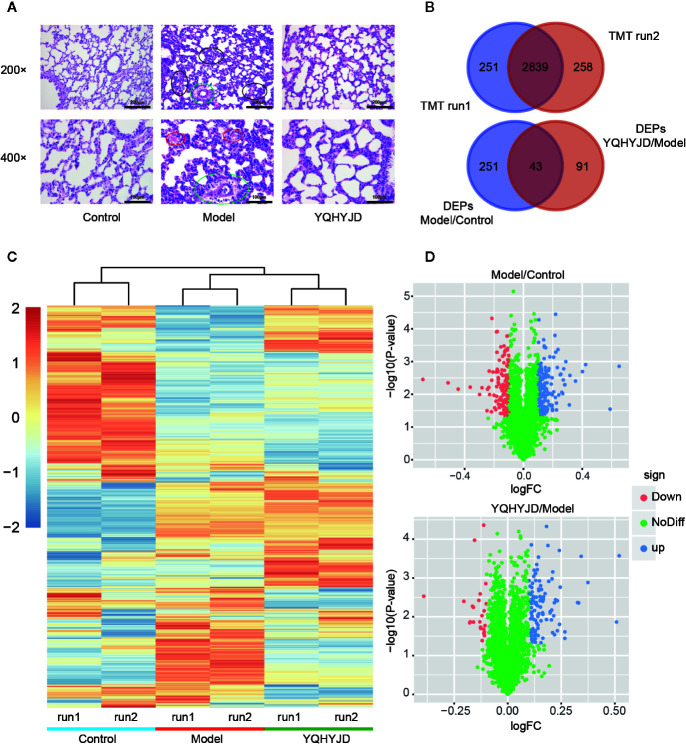
**(A)** The lung tissues were subjected to hematoxylin and eosin staining and observed under a light microscope at 200X and 400X magnifications. The representative images of each group are shown at scale bars of 200 and 100 µm. Normal control group (Control); acute respiratory distress syndrome model control group (Model); TCM, Yiqi Huayu Jiedu Decoction (YQHYJD) treatment group. The black circle indicates edema of alveoli and interstitium, infiltration of inflammatory cells, thickening of alveolar wall, atrophy, and deformation of alveolar cavity, and collapse of some alveoli. The red circles indicate the presence of red blood cells in the alveoli, the green circle indicates congestion and edema of pulmonary capillaries. **(B)** The results of two tandem mass tag (TMT) protein labeling experiments. The red part TMT1 is the result of first protein labeling, while the blue part TMT2 is the result of second protein labeling, and the deep red intersection part is the overlap between two protein labeling. **(C)** Cluster heat map of each protein expression. Control group; 1.1, TMT1 labeling, 1.2, TMT2 labeling, Model, acute respiratory distress syndrome model control group; YQHYJD, Yiqi Huayu Jiedu Decoction (YQHYJD) treatment group. Upregulated protein expression is indicated in red, while the downregulated protein expression is indicated in blue. **(D)** The volcano map of the Model group compared to the Control group and YQHYJD group compared with the Model group. The log_2_fold-change (FC) value is the abscissa and the -Log_10_(P-value) is the ordinate. UP blue indicates the differentially expressed proteins that are upregulated, while the Down red indicates those that are downregulated. The NoDiff green indicates the protein with no significant change in expression.

The pathological changes in the lung tissue and the protective effect of YQHYJD in ARDS rats were evaluated by hematoxylin and eosin staining (**Figure 3A**). The Control group lung tissue exhibited clear and intact alveolar structure with no edema of alveolar septum, no tissue fluid in the alveolar cavity, no inflammatory cell infiltration in the lung tissue. The Model group lung tissue exhibited alveolar and interstitial edema with a large amount of inflammatory cell infiltration, alveolar wall thickening and visible alveolar cavity shrinkage and deformation, partial alveolar collapse, secretions containing red blood cells in the bronchial and alveolar cavities, pulmonary capillary congestion, and edema. Treatment with YQHYJD alleviated the lung tissue damage with less inflammatory cell infiltration and alveolar collapse, decreased bleeding and exudation, and less severe pulmonary microvascular endothelial edema.h

### Effect of YQHYJD on Protein Expression in the Lung Tissue of ARDS Rats

The sodium dodecyl sulfate-polyacrylamide gel electrophoresis analysis revealed that the samples in each group were clear and the proteins resolved with no obvious diffusion and deformation. The protein aggregation sites were similar and evenly distributed, which indicated that the samples were comparable. After two TMT-labeled replicate experiments, 3,090 proteins were identified in the first round and 3,097 proteins were identified in the second round. In total, 3,348 proteins were identified and quantified. Of these, there were 2,839 common proteins (**Figure 3B**). Hierarchical cluster analysis revealed that the samples clustered into the following three groups: Control, Model, and YQHYJD groups (**Figure 3C**). There was little intra-group variability and high inter-group comparability. Compared to the Control group, 294 DEPs were identified in the Model group (p < 0.05 and log FC > 0.1). Of these 294 DEPs, 131 were down-regulated and 163 were up-regulated. Compared to the Model group, 134 DEPs were detected in the YQHYJD group. Of these 134 DEPs, 24 were down-regulated and 110 were up-regulated (**Figure 3D**). See **Table S1** for total protein expression data. Specifically, 10 proteins were up-regulated by LPS-induced ARDS, but down-regulated by YQHYJD (Set 1), and 22 proteins were down-regulated by LPS-induced ARDS, but up-regulated by YQHYJD (Set 2). Also, the proteins showing no changes by LPS-induced ARDS, but up-regulated (Set 3) or down-regulated (Set 4) by YQHYJD can augment the therapeutic effect of YQHYJD. 87 proteins showed the augmenting patterns (**Figure S1**).

### Gene Ontology (GO) Term Enrichment and Kyoto Encyclopedia of Genes and Genomes (KEGG) Pathway Enrichment Analyses

**Figure 4:**
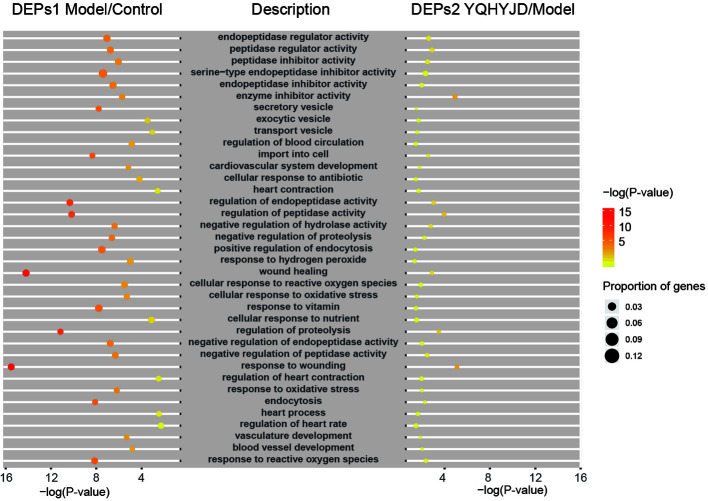
It showed common GO terms. The size of the dot represents the number of gene proportion and the color represents the P-value. The yellower the color indicates the smaller the –log(P-value), and the redder the color indicates the larger the –log(P-value).

Both the DEPs 1 of Model and Control groups and DEPs 2 of YQHYJD and Model groups were subjected to GO term enrichment analysis, which included Biological Process, Cellular Component, and Molecular Function terms. There are 37 GO enrichment functions in common between DEPs 1 and DEPs 2 (**Figure 4**). In addition, we also showed the unique top 20 GO enrichment functions of DEPs1 and DEPs2 in **Figure S2** and **Table S2**. The top 10 common GO terms were response to wounding, wound healing, regulation of proteolysis, regulation of endopeptidase activity, regulation of peptidase activity, import into cell, response to reactive oxygen species, endocytosis, response to vitamin, positive regulation of endocytosis. Enrichment analysis was also carried out for each set. Set 1 and Set 2 were enriched to few significant biological functions, which may be related to the small number of differential proteins. Set 3 showed that traditional Chinese medicine might be related to biological functions such as extract matrix, actin fixation, vascular membrane, platelet-derived growth factor binding, and so on. Set 4 showed that traditional Chinese medicine might play a role in nucleotide biosynthesis and metabolism, negative regulation of nervous system, cardiovascular system development and so on. The results suggest that Chinese medicine can play a certain therapeutic role by enhancing the curative effect (**Table S3**).

**Figure 5:**
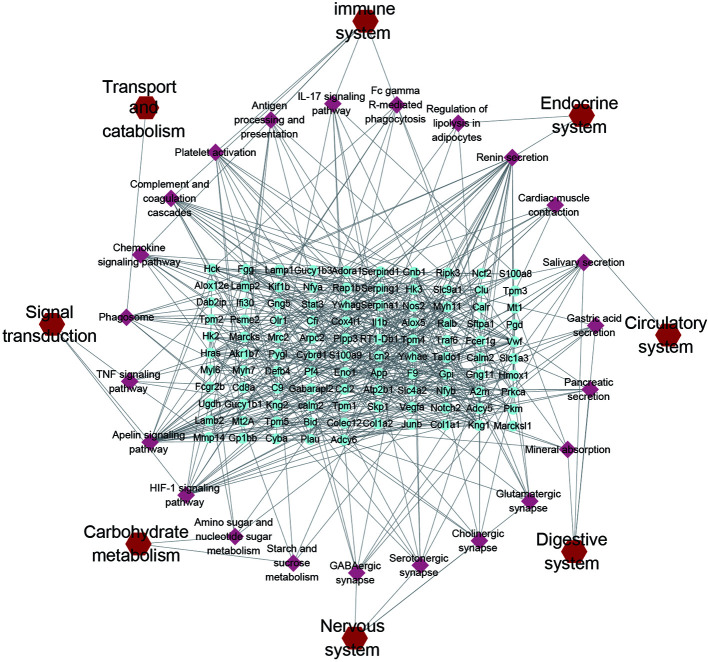
KEGG pathways network. The blue plots represent the DEPs, the pink plots represent the pathways, and the red plots represent the types of pathways.

All DEPs were used for KEGG pathway analysis, pathways with -log(P-value) greater than 2 and gene proportion greater than 5% were screened out, the results show that there were 23 pathways, they are related to the immune system, endocrine system, digestive system, circulatory system, nervous system, Signal transduction, carbohydrate metabolism and transport and catabolism. The number of immune system related pathways and genes is the largest. The networks of KEGG pathway types, KEGG pathways and genes are constructed in Cytoscape (**Figure 5**). The specific pathways’ P-value and gene proportion are shown in the **Table S4**. The results of KEGG pathway analysis of each set showed that only a few meaningful pathways were obtained by Set 3, which were ECM receptor interaction, platelet activation, focal adhesion, cardiomyopathy, and so on (**Table S3**).

### PRM Validation of Proteins Affected by YQHYJD Treatment in ARDS Rats

**Figure 6:**
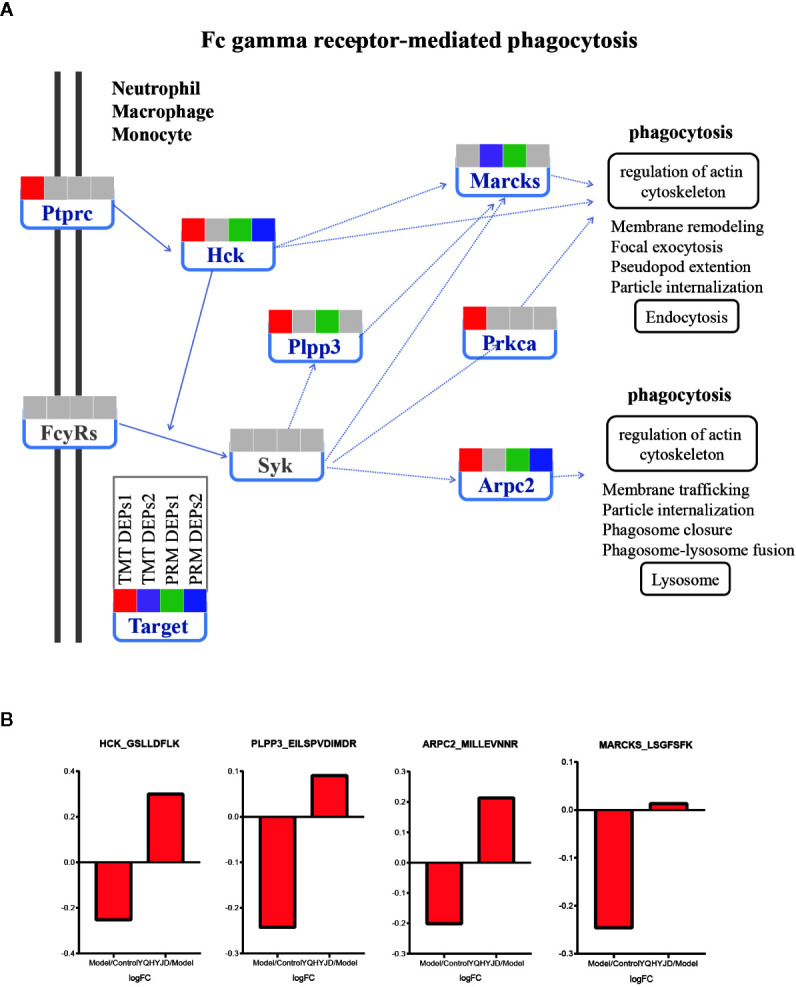
Expression of TMT DEPs and PRM verification in Fc gamma receptor-mediated phagocytosis pathway. **(A)** Intervention of YQHYJD on Fc gamma receptor-mediated phagocytosis pathway. **(B)** Verification of intervention effect of YQHYJD on protein Hck, Plpp3, Marcks, and Arpc2.

Based on proteomic analysis, Immune system related pathways were taken as the key research object. The PRM method was used to verify the targets involved in immune related pathways, and eight proteins obtained significant results. Hematopoietic cell kinase (Hck), phospholipid phosphatase 3 (Plpp3), myristoylated-alanine rich C-kinase substrate (Marcks) and Actin-related protein 2/3 complex subunit 2 (Arpc2) down regulated in the Model group, alleviated in YQHYJD group (**Figure 6**). In KEGG pathways, these proteins belong to Fc gamma receptor-mediated phagocytosis pathway (**Figure 5**), which suggests that in the model group, the phagocytosis of cells is inhibited, and YQHYJD could increase the phagocytosis of these cells and played the role of resistance to external microorganisms. In addition, the expression of collagen I (Col1a1) and Ras-related protein Rap1b decreased in the model group, which could be improved by YQHYJD (**Figure S3**), Col1a1 and Rap1b proteins belong to Platelet activation pathway In KEGG pathways (**Figure 5**), suggesting that YQHYJD may promote platelet activation, so as to improve the hypercoagulable state of blood in the lungs of animals with ARDS. The results of GTPase Hras and Lipocalin-2 (Lcn2) are also significant (**Figure S3**). They participated in IL-17 signaling pathway and chemokine signaling pathway. KEGG showed that both pathways could promote neutrophil migration, suggesting that YQHYJD may have an intervention effect in this respect. The TMT and PRM results of the eight target proteins verified were shown in **Table S5**.

## Discussion

Since the first description of ARDS in 1967, there has been little progress in developing novel therapies for ARDS. Currently, there are no effective therapeutic agents for preventing ARDS or alleviating its symptoms. Thus, the morbidity and mortality of patients with ARDS remain high (Boyle et al., 2013; Impellizzeri et al., 2015; Pham and Rubenfeld, 2017). Recently, a consensus-based Berlin definition proposed to subdivide ARDS into three categories based on the degree of hypoxemia. Furthermore, it was suggested that the term “acute lung injury” should be dropped by the practitioners. In a recent international study, sepsis was reported to be a potential cause of ARDS in approximately 75% patients (59% pneumonia and 16% extrapulmonary sepsis) (Ranieri et al., 2012; Sweatt and Levitt, 2014; Bellani et al., 2016). In the United States, sepsis is estimated to cause ARDS in more than 210,000 patients each year (Rubenfeld et al., 2005; US and World Population Clock and US and World Population Estimates, 2018). The mortality of patients with ARDS induced by sepsis is higher than that induced by other factors (Stapleton et al., 2015). In this study, an animal model of ARDS was generated by injecting LPS into the tail vein. The pathological features of the lung in this model were consistent with the characteristics of ARDS.

ARDS occurs due to aberrant acute inflammation and dysfunction of endothelial and epithelial barriers of the lung and excessive transepithelial leukocyte migration. This results in the loss of alveolar capillary membrane integrity and the overproduction of proinflammatory cytokines. The pathogenesis of ARDS involves the activation of both immune and structural cell types. The major immune cells involved in ARDS are neutrophils, macrophages, lymphocytes, and platelets (Fanelli et al., 2013; Butt et al., 2016; Confalonieri et al., 2017). The pathogenesis of sepsis and ARDS is closely related to the aberrant activation of inflammatory responses, which play a key role in the development of ARDS (Wohlrab et al., 2015).

Previously, we had demonstrated that YQHYJD not only downregulates the expression of proinflammatory pathway proteins and proinflammatory factors but also increases the expression level of anti-inflammatory factors (Luo et al., 2019). This suggested that YQHYJD protects the lung tissue of ARDS rats by modulating the balance between proinflammatory and anti-inflammatory factors. In this study, the lung tissue proteins of each experimental group were labeled with TMT. The proteins that are differentially expressed upon YQHYJD intervention were subjected to bioinformatics analysis. The following proteins recovered after treatment with YQHYJD: Hck, Plpp3, Marcks, Arpc2, Col1a1, Rap1b, Hras, and Lcn2. In the KEGG pathway database, these proteins are related to phagocytosis, platelet activation and neutrophil migration. Similarly, the results of phagocytosis and platelet activation of these proteins were also reported. Studies have shown that Hck deficiency weakens the phagocytic function of microglia a β (Lim et al., 2020). Hck is one of the key regulators of phagocytosis among the Src family tyrosine kinases (SFKs) in myeloid cells and combined absence of the Src family kinases Hck drastically impairs phagocytosis (Lim et al., 2018; Lőrincz et al., 2019). Drugs could inhibit Marcks expression, consequently, blocking Fc gamma receptor-mediated phagocytosis pathway, which contributed to the cancer progression *in vitro* and *in vivo* (Luo et al., 2019). Study reveals that the Arpc2 is not a general requirement for phagocytosis or chemotaxis but is a critical driver of integrin-dependent processes (Rotty et al., 2017). Rap1b was critical for platelet functions and was the main aggregation point of platelet signaling pathway, and played an important role in regulating platelet integrin activation during hemostasis (Dahiya and Atreya, 2020; Lagarrigue et al., 2020).

In this study, through TMT proteomics and PRM verification methods, it was clear that the protein of Hck, PPlpp3, Marcks and Arpc2 was significantly reduced in ARDS model, which indicated that the phagocytosis of cells in the lung of model animals was reduced. A multicenter clinical study has found that the phagocytosis dysfunction of alveolar macrophages in ARDS patients is related to the severity of the disease. In animal studies, enhancing the phagocytosis of alveolar macrophages and monocytes *in vivo* can reduce the degree of lung inflammation damage in model animals (Jackson et al., 2016; Du et al., 2019). The decrease of neutrophil phagocytosis in Bronchoalveolar lavage fluid (BALF) of patients with ARDS will also decrease the bacterial clearance rate and increase the pulmonary sepsis caused by ARDS (Griswold and Maier, 1988). The results showed that YQHYJD could up regulate the expression of Hck and Arpc2, suggesting that YQHYJD might play an anti-microbial role by regulating the phagocytosis of cells, so as to reduce the inflammatory damage of lung tissue. A number of studies have shown that coagulation dysfunction is closely related to prognosis in patients with ARDS caused by antigens such as bacteria and viruses (Wu et al., 2020). In critical patients, microthrombosis and thrombocytopenia coexist. The worsening thrombocytopenia is the most important laboratory sign suggesting progression of ARDS. In this situation, platelet transfusion might be tempting, but it is contraindicated in endotheliopathy-associated vascular microthrombotic disease because platelet transfusion would further promote microthrombogenesis (Chang et al., 2019). The produced platelet-neutrophil complexes participate in the defense mechanism by increasing phagocytosis capacity (Frantzeskaki et al., 2017). In this study, YQHYJD can increase the expression of Col1a1 and Rap1b, which are involved in platelet activation. In summary, YQHYJD plays a role in reducing lung injury by participating in multiple immune related pathways such as Fc gamma receptor-mediated phagocytosis. Further studies are needed to elucidate the therapeutic mechanism of YQHYJD for lung injury *in vitro*. Meanwhile, it is known that high mortality of ARDS has correlation with not only the damaged lung, but also the failure of extrapulmonary organs. YQHYJD can alleviate liver swelling and intestinal edema in rats with LPS-induce ARDS, which may be related to the multi-target and complementary mechanism of traditional Chinese medicine compound, which is worthy of further study in the future.

## Conclusion

YQHYJD can alleviate the LPS-induced pathological damage to lung tissue of rats. The analysis pathway enrichment of DEPs showed that the pathways of the immune system play ed an important role in ARDS. Hck, Plpp3, Marcks, and Arpc2 verified by PRM. These proteins are related to KEGG pathways and biological function, such as Fc gamma receptor-mediated phagocytosis, which are required for the protective effect of YQHYJD against lung tissue damage.

## Data Availability Statement

The datasets presented in this study can be found in the supplementary materials (**Supplementary Table S1–S5** and **Figure S1–S3**) and online repository iProX, with accession ID IPX0002375000 (http://www.iprox.org).

## Ethics Statement

The experimental animal research has been approved by the Experimental Animal Welfare and Ethics Committee of Dongzhimen Hospital of Beijing University of Chinese Medicine (batch number: 17-08).

## Author Contributions

C-YL and XiL designed and conducted the study with equal contribution. C-YL, XiL, XuL, QW, Y-HM, C-HX, and Y-PZ performed the experiments. C-YL, WS, and XW analyzed the data. C-YL and YL prepared the manuscript. All authors contributed to the article and approved the submitted version.

## Funding

This work was supported by grants from the Horizontal Subject of Dongzhimen Hospital, Beijing University of Chinese Medicine (HX-DZM-2017005). The authors declare that they have no conflicts of interest with the contents of this article.

## Conflict of Interest

The authors declare that the research was conducted in the absence of any commercial or financial relationships that could be construed as a potential conflict of interest.

## Supplementary Material

Table S1 Total protein expression data of TMT test.

Table S2 Unique top 20 Gene Ontology (GO) term enrichment of DEPs1 in sheet of unique1. Unique top 20 GO enrichment functions of DEPs2 in sheet of unique2.

Table S3 The results of GO term enrichment and Kyoto Encyclopedia of Genes and Genomes (KEGG) pathways in each set.

Table S4 All 23 meaningful KEGG pathways.

Table S5 PRM Verification of intervention effect of YQHYJD on protein Hck, Plpp3, Marcks, Arpc2, Rap1b, Col1a1, Hras, and Lcn2.

Figure S1 Red means up regulation; blue means down regulation. Close to white means that the change of logFC is not significant.10 proteins (Set 1) were up-regulated by LPS-induced ARDS, but down-regulated by YQHYJD. 22 proteins (Set 2) were down-regulated by LPS-induced ARDS, but up-regulated by YQHYJD. The proteins showing no changes by LPS-induced ARDS, but 74 proteins (Set 3) up-regulated by YQHYJD. The proteins showing no changes by LPS-induced ARDS, but 13 proteins (Set 4) down-regulated by YQHYJD.

Figure S2 Unique top 20 GO enrichment functions of DEPs1 and DEPs2 were showed. The size of the dot represents the number of Gene proportion; the color represents the value of P-value. The yellower the color, the smaller the -log(P-value), the larger the P-value and the redder the color, the larger the -log(P-value), the smaller the P-value.

Figure S3 Verification of intervention effect of YQHYJD on protein Rap1b, Col1a1, Hras, Lcn2.

## Abbreviations

ARDS, Acute respiratory distress syndrome; TCM, traditional Chinese medicine; YQHYJD, Yiqi Huayu Jiedu Decoction; NF-κB, nuclear factor kappa-B; TNF-α, tumor necrosis factor α; LPS, Lipopolysaccharide; IL, interleukin; DEPs, differentially expressed proteins; TMT, tandem mass tag; NC, normal control; MC, model control; PRM, Parallel reaction monitoring; PPI, protein-protein interaction; GO, Gene Ontology; KEGG, Kyoto Encyclopedia of Genes and Genomes.
