# Supplementary figures and images for: Alleviation of Lipopolysaccharide-Induced Acute Respiratory Distress Syndrome in Rats by Yiqi Huayu Jiedu Decoction: A Tandem Mass Tag-Based Proteomics Study
Source: Front Pharmacol. 2020 Aug 28;11:1215. doi: 10.3389/fphar.2020.01215 (PMC7485520; doi:10.3389/fphar.2020.01215)

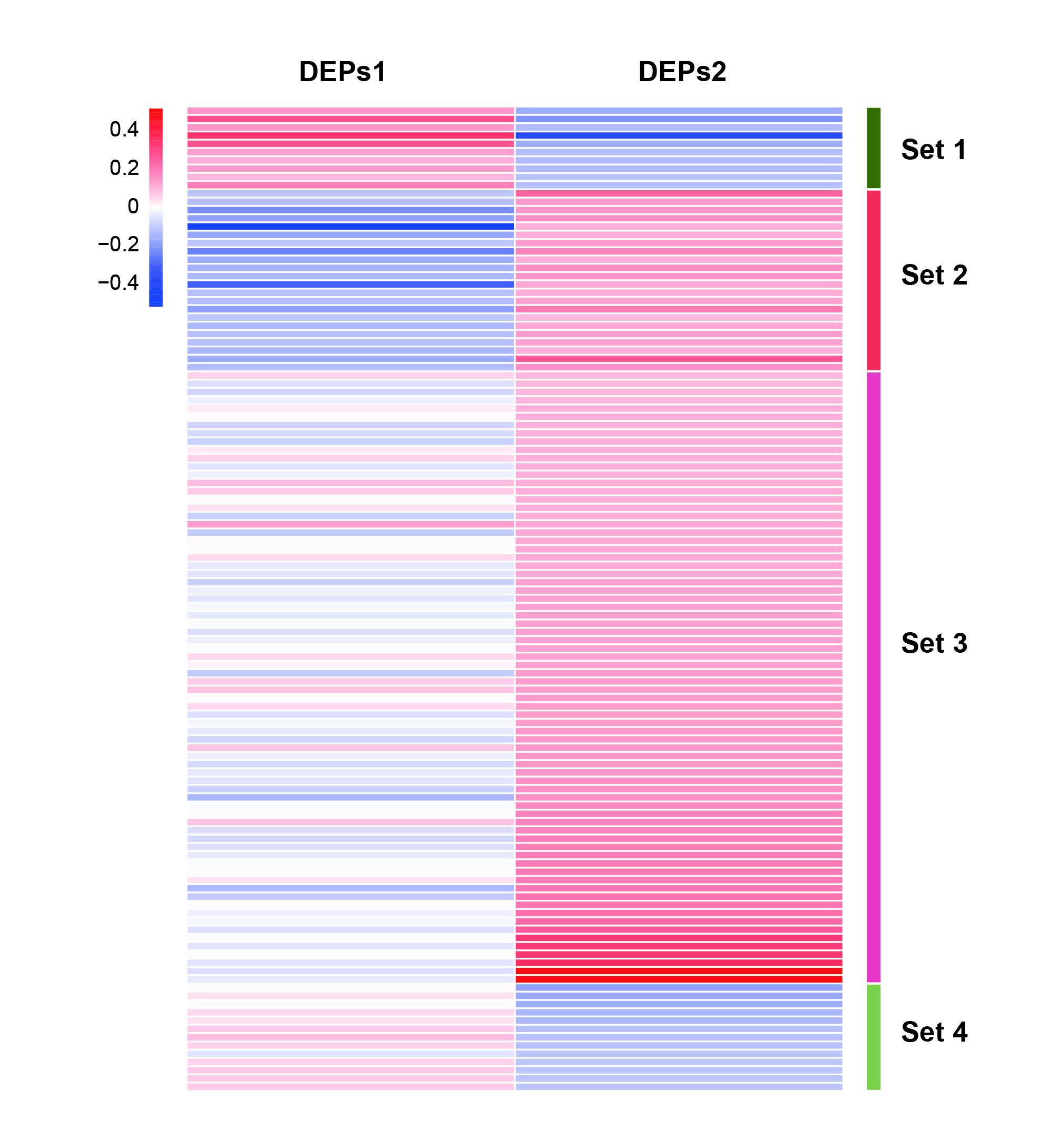

Supplement: Figure S1 — Red means up regulation; blue means down regulation. Close to white means that the change of logFC is not significant.10 proteins (Set 1) were up-regulated by LPS-induced ARDS, but down-regulated by YQHYJD. 22 proteins (Set 2) were down-regulated by LPS-induced ARDS, but up-regulated by YQHYJD. The proteins showing no changes by LPS-induced ARDS, but 74 proteins (Set 3) up-regulated by YQHYJD. The proteins showing no changes by LPS-induced ARDS, but 13 proteins (Set 4) down-regulated by YQHYJD. [file Image_1.jpeg]

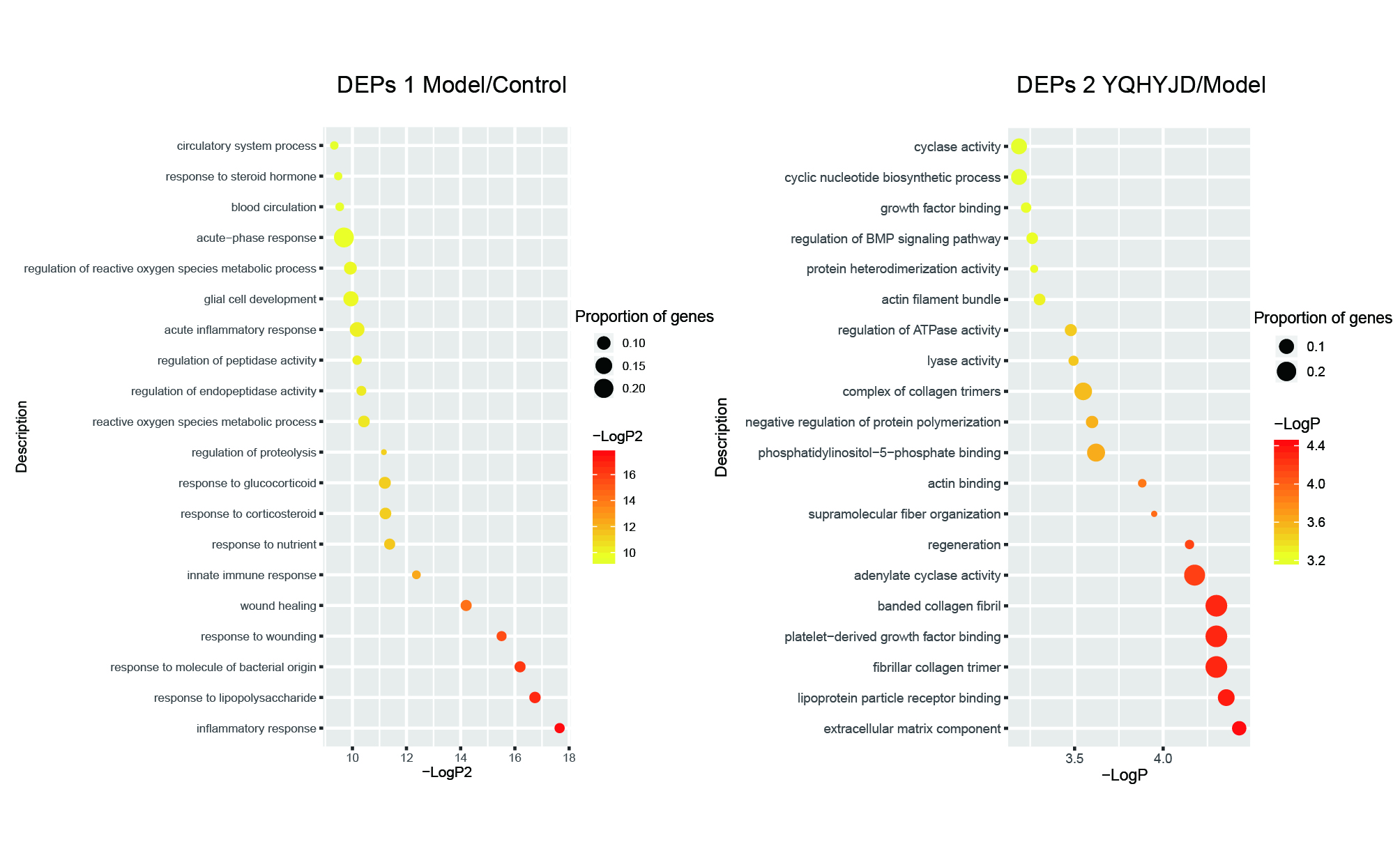

Supplement: Figure S2 — Unique top 20 GO enrichment functions of DEPs1 and DEPs2 were showed. The size of the dot represents the number of Gene proportion; the color represents the value of P-value. The yellower the color, the smaller the -log(P-value), the larger the P-value and the redder the color, the larger the -log(P-value), the smaller the P-value. [file Image_2.jpeg]

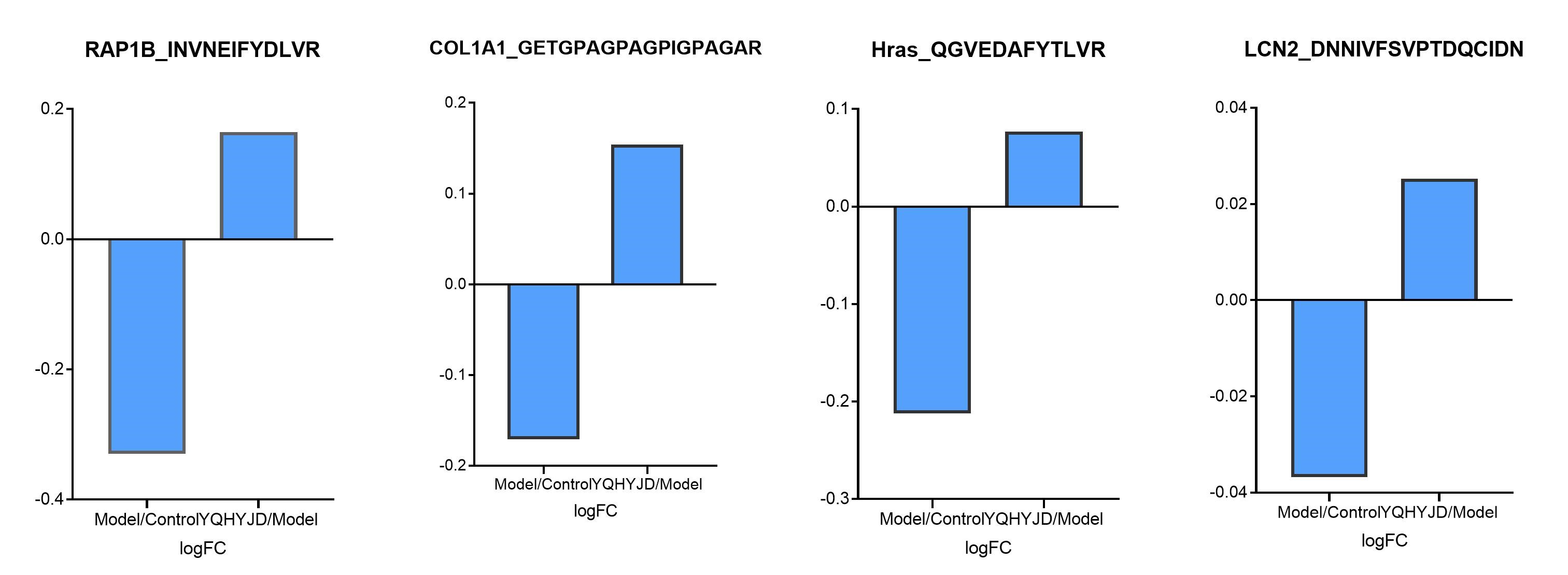

Supplement: Figure S3 — Verification of intervention effect of YQHYJD on protein Rap1b, Col1a1, Hras, Lcn2. [file Image_3.jpeg]
